# Identification of candidate miRNAs in early-onset and late-onset prostate cancer by network analysis

**DOI:** 10.1038/s41598-020-69290-7

**Published:** 2020-07-23

**Authors:** Rafael Parra-Medina, Liliana López-Kleine, Sandra Ramírez-Clavijo, César Payán-Gómez

**Affiliations:** 1 Department of Biology, Faculty of Natural Sciences, Universidad del Rosario, Bogotá, Colombia; 2 Department of Pathology, Research Institute, Fundación Universitaria de Ciencias de la Salud, Bogotá, Colombia; 3 Pathology Deparment, Instituto Nacional de Cancerología, Bogotá, Colombia; 4 Department of Statistics, Faculty of Science, Universidad Nacional de Colombia, Bogotá, Colombia

**Keywords:** Prostate cancer, miRNAs

## Abstract

The incidence of patients under 55 years old diagnosed with Prostate Cancer (EO-PCa) has increased during recent years. The molecular biology of PCa cancer in this group of patients remains unclear. Here, we applied weighted gene coexpression network analysis of the expression of miRNAs from 24 EO-PCa patients (38–45 years) and 25 late-onset PCa patients (LO-PCa, 71–74 years) to identify key miRNAs in EO-PCa patients. In total, 69 differentially expressed miRNAs were identified. Specifically, 26 and 14 miRNAs were exclusively deregulated in young and elderly patients, respectively, and 29 miRNAs were shared. We identified 20 hub miRNAs for the network built for EO-PCa. Six of these hub miRNAs exhibited prognostic significance in relapse‐free or overall survival. Additionally, two of the hub miRNAs were coexpressed with mRNAs of genes previously identified as deregulated in EO-PCa and in the most aggressive forms of PCa in African-American patients compared with Caucasian patients. These genes are involved in activation of immune response pathways, increased rates of metastasis and poor prognosis in PCa patients. In conclusion, our analysis identified miRNAs that are potentially important in the molecular pathology of EO-PCa. These genes may serve as biomarkers in EO-PCa and as possible therapeutic targets.

## Introduction

The incidence of patients under 55 years old diagnosed with prostate cancer (PCa) (Early onset, EO-PCa) in the United States has increased during recent years. Between 1986 and 2008, the incidence of EO-PCa was from 5.6 to 32 cases per 100.00 persons years (IC 95% CI 5.0–6.7)^1,2^. In 2012, PCa was diagnosed in 241,740 men (10%) < 55 years old in the United States^3^. Thus, PCa in young patients is an emerging issue for public health^1,2^. Interest in understanding the molecular and clinical behavior of EO-PCa has been increased^4^. Several risk factors are associated with diagnosis: family’s medical background, ethnicity, and genetic factors, such as single nucleotide polymorphisms and mutations in BRCA1, BRCA2, and HOXB13^5,6^. Different single nucleotide polymorphisms in germinal DNA^7^ and rearranged genes in the androgen receptor axis (e.g., TMPRSS2-ERG, PTEN, and AR) have been identified EO-PCa^8^. Additionally, abnormal expression of genes involved in inflammatory and antitumoral immune-related pathways (CTL4, IDO1/TDO2) was detected^9^. A recent analysis of 1281 EO-PCa cases (≤ 60 years) identified 23 unique DNA repair genes associated with an increased predisposition or risk of aggressive PCa disease, and four genes (BRCA2, MSH2, ERCC2, and CHEK2_non1100del) were associated with more aggressive disease^10^. Other recent studies identified four molecular subgroups that included a particularly aggressive subgroup with recurrent duplications (8q22) associated with increased ESRP1 expression^11^.

MicroRNAs (miRNAs) are small (~ 20–22 nucleotides), noncoding RNA molecules that are well conserved among different species of organisms and play multiple roles in several biological processes. miRNAs can interact with the RNAm of their target gene to exert its biological regulatory effect on gene expression by inhibiting the translation process^12^. This effect is achieved by binding to the cognate sequence 3′ UTR of RNAm to promote its degradation or inhibit the translation process^13^. Transcription activation is a non-canonical mechanism of miRNA action that was recently described^14^. In addition, miRNAs regulate expression of up to 30% of human genes with a proven impact in significant parts of different molecular pathways, including cell proliferation, differentiation and apoptosis^13^. In addition, miRNA expression in human cancer is dysregulated as a result of chromosomal disorder (amplification, translocations, and deletions), the presence of single-nucleotide polymorphisms (SNPs), the induction of epigenetic changes, deficiency in its biogenesis machinery, and modification of the expression of transcription factors necessary to control miRNA gene transcription^15^.

Different miRNAs may have similar expression profiles when the miRNAs are functionally related or modulate the same pathway^16^. Based on the previous assumption, the Weighted Gene Coexpression Network Analysis (WGCNA) has been used to calculate the level of correlation among miRNA expression and identify clusters of coexpressed miRNAs in biological samples. A cluster of coexpressed miRNAs could be involved in the same pathway or biological process^17^. Additionally, using network analysis of coexpressed miRNA clusters, it is possible to identify its most central point within a cluster, namely, the hub genes, that could play the most important role or function in the initial step in the deregulation of other miRNAs.

In PCa, numerous miRNAs involved in different process related to PCa oncogenesis, such as cell cycle, apoptosis, epithelial-mesenchymal transition, DNA replication/repair, migration, androgen receptor suppression, metastasis, and treatment resistance, have been described^18^. Therefore, the miRNAs are studied as promising candidates that can be detected using minimally invasive diagnostic techniques and prognostic biomarker tools^19^. Several miRNAs involved in tumor growth are upregulated and downregulated in recurrent PCa compared to nonrecurrent PCa samples^20^. In EO-PCa patients, miRNA expression has been evaluated in a few studies^8,9,21^ and differences in expression profiles have been observed compared to LO-PCa (Late onset-PCa). Weischenfeldt et al.^8^ focused the analysis on miRNAs involved in the PTEN pathway. Some upregulated and downregulated miRNAs were detected, and some of genes with hypermethylated promoter regions, particularly tumor suppressor genes, exhibit reduced expression (hsa-miR-106b-5p, hsa-miR-93-5p, hsa-miR-25-3p, hsa-miR-141-3p). Ding et al.^9^ found several miRNAs with deregulated expression (miRNAs DE); however, the analysis focused on mRNAs and found genes mainly involved in inflammation pathways. Recently, Valera et al.^21^ found miRNAs DE in EO-PCa tumor tissue compared to LO-PCa as well as tumor tissue compared to normal tissue. They also identified miRNAs associated with high Gleason score, extraprostatic extension and lymphatic invasion. In these studies, deeper miRNA expression analyses were not performed. Therefore, we employed a systems biology analysis to identify fundamental miRNAs with transcriptional alterations, their target genes and coexpressed mRNAs that can explain the early appearance of PCa as well as the increased aggressiveness and different responses to treatment noted in these tumors.

## Results

### Data selection

Database analyses identified 3623 articles, of which 506 were duplicates. In total, 5 full-text articles were assessed for eligibility, and one paper met the inclusion criteria: GSE89193^9^. The pathological stage of all tumors was T2 (T2a and T2c), and the Gleason score was 7 (3 + 4). Among these patients, 67% had PSA ≤ 10.0. In total, 58% and 76% of young and old patients, respectively, had PSA ≤ 10.0 In both groups, 88% (n = 22) were white, 4% (n = 1) African-Americans, 4% (n = 1) Hispanics and 4% (n = 1) Asians.

Total RNA was extracted from the primary tumor tissue and matched control normal tissue samples, which were obtained from formalin-fixed paraffin-embedded tissue blocks from prostatectomies. The small RNA profile was generated using the Illumina Human Whole-Genome DASL (cDNA-mediated annealing, selection, extension, and ligation), while the miRNAs were sequenced on the Illumina HuSeq 2500 platform.

### Identification of differentially expressed miRNAs

**Figure 1:**
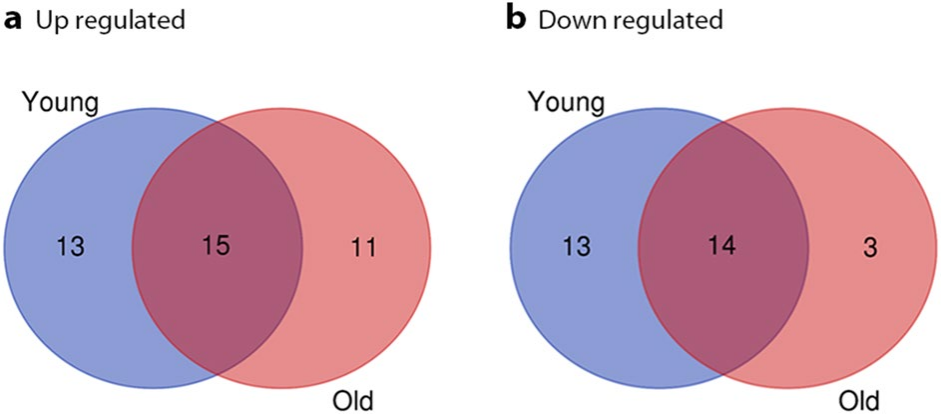
Venn diagram of DE-miRNAs. A. Upregulated DE-miRNAs; B. Downregulated DE-miRNAs.

The comparison between transcriptomes of tumor and normal prostate samples employed stringent criteria of a fold change (FC) greater than 2 and less than -2 and a false discovery rate (FDR) less than 0.01. In the LO-PCa group, 43 miRNAs were identified as differentially expressed, including 26 upregulated and 17 downregulated miRNAs. In the EO-PCa group, 55 miRNAs were identified as differentially expressed, including 28 upregulated and 27 downregulated miRNAs. Subsequently, the two lists of differentially expressed miRNAs included 69 miRNAs DE in total with 29 miRNAs in common, and further analyses were conducted (Fig. 1 and Supplementary Table 1).

**Table 1:** Top 20 of hub miRNAs in young and old obtained from CytoHubba analysis.

Hub miRNAs in young					Hub miRNAs in old				
Rank	Name	Score MCC	logFC	adj.P.Val	Rank	Name	Score MCC	logFC	adj.*P*.Val
1	hsa-miR-31-5p	7716	− 2.021	0.00001	1	hsa-miR-32-5p	182	1.372	0.00007
2	hsa-miR-224-5p	7705	− 1.052	0.00000	2	hsa-miR-96-5p	169	1.216	0.00006
3	hsa-miR-3065-3p	744	− 1.082	0.00051	3	hsa-miR-182-3p	168	1.007	0.00942
4	hsa-miR-205-5p	6858	− 2.804	0.00002	4	hsa-miR-183-5p	160	0.986	0.00000
5	hsa-miR-205-3p	6738	− 2.283	0.00003	5	hsa-miR-375	156	1.297	0.00000
6	hsa-miR-3545-3p	6368	− 2.509	0.00000	6	hsa-miR-183-3p	126	0.886	0.00454
7	hsa-miR-224-3p	5544	− 1.052	0.00000	7	hsa-miR-224-3p	90	− 1.066	0.00039
8	hsa-miR-676-3p	5424	− 1.249	0.00024	8	hsa-miR-205-5p	80	− 2.327	0.00000
9	hsa-miR-135b-5p	1680	− 1.951	0.00161	9	hsa-miR-224-5p	72	− 1.142	0.00000
10	hsa-miR-452-3p	1560	− 0.761	0.00090	10	hsa-miR-31-5p	60	− 1.813	0.00018
11	hsa-miR-488-3p	846	− 1.368	0.00358	11	hsa-miR-3545-3p	48	− 1.829	0.00012
12	hsa-miR-1911-5p	762	− 2.578	0.00027	12	hsa-miR-452-5p	42	− 1.014	0.00000
13	hsa-miR-1912	732	− 2.022	0.00228	13	hsa-miR-32-3p	36	1.101	0.00160
14	hsa-miR-509–3-5p	258	− 2.235	0.00032	14	hsa-miR-1298	24	− 1.855	0.07505
15	hsa-miR-31-3p	246	− 1.557	0.00157	15	hsa-miR-10a-3p	24	− 0.391	0.29418
16	hsa-miR-452-5p	244	− 0.974	0.00000	16	hsa-miR-5096	24	1.181	0.00334
17	hsa-miR-150-5p	26	1.120	0.00005	17	hsa-miR-1911-5p	24	− 1.032	0.19284
18	hsa-miR-142-5p	26	1.140	0.00008	18	hsa-miR-1912	24	NS	NS
19	hsa-miR-146b-3p	25	1.568	0.00000	19	hsa-miR-205-3p	24	− 1.942	0.00006
20	hsa-miR-514a-3p	24	− 2.516	0.00008	20	hsa-miR-944	18	− 1.988	0.00000

### Functional enrichment analysis of miRNAs with dysregulated expression

**Figure 2:**
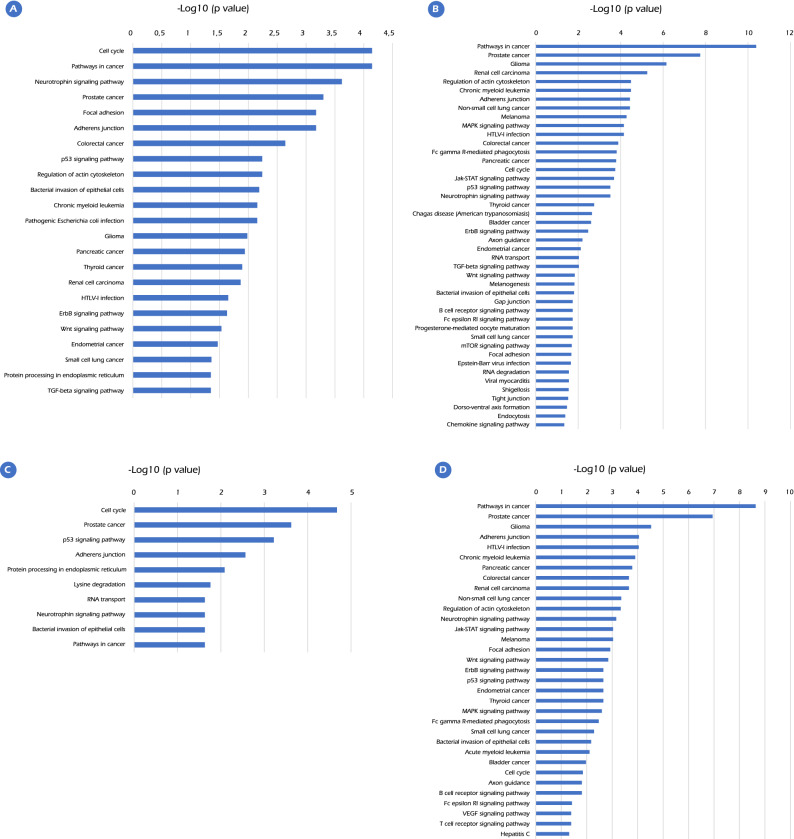
Pathway enrichment analysis for the predicted target genes of potential DE-miRNAs (*p* value < 0.05). (**A**). Enriched KEGG pathways for target genes of upregulated DE-miRNAs in EO-PCa. (**B**). Enriched KEGG pathways for target genes of downregulated DE-miRNAs in EO-PCa. (**C**). Enriched KEGG pathways for target genes of upregulated DE-miRNAs in LO-PCa. (**D**). Enriched KEGG pathways for target genes of downregulated DE-miRNAs in LO-PCa.

KEGG pathway enrichment analysis was successively predicted by miRNet and aimed to validate that these miRNAs DE are involved in the prostate cancer pathway. This analysis revealed that in EO-PCa samples, 23 and 44 pathways were present in upregulated and downregulated miRNAs, respectively. In LO-PCa, 10 and 33 pathways were upregulated and 33 downregulated, respectively (Fig. 2). Genes in the PCa pathway as annotated by KEGG were overrepresented as targets in upregulated and downregulated miRNAs in EO-PCa and LO-PCa. The detected pathways are dysregulated by upregulated and downregulated miRNAs in EO-PCa and by downregulated miRNAs in LO-PCa. These pathways had roles in carcinogenesis, such as increasing the cellular proliferation rate, reducing cellular focal adhesion, and alteration of signaling pathways, such as MAPK, p53, Jak-STAT, neurotrophin, Wnt and ErbB. These results are similar to previous reports in renal cell carcinoma and thyroid cancer. The identified pathways dysregulated by upregulated miRNAs in LO-PCa included cellular proliferation, p53 signaling pathway, protein processing in the endoplasmic reticulum, adherens junction formation, and amino acid lysine degradation. Supplementary File 1 shows the target genes present in each of the dysregulated pathways.

### Weighted coexpression networks

To capture most biological differences in the compared groups, we used less stringent criteria to select differentially expressed miRNAs: FC greater than 1.5 or less than – 1.5 and FDR less than 0.05. With those parameters, we identified in total 157 miRNAs differentially expressed (DE), 102 miRNAs DE in EO-PCa, 121 miRNAs DE, and 66 miRNAs DE in common. Based on the expression levels of the 157 miRNAs DE, two coexpression networks were calculated. To generate comparable Weighted Coexpression Networks (WGCA), we used the same list of miRNAs in each of the two networks generated. First, the 24 samples from young patients were used, and the similarity threshold was calculated using the maximum local method. A Pearson correlation coefficient greater than or equal to 0.57 differentiates the distributions of the correlations between the miRNAs and those of a random population. In total, 35 miRNAs had a correlation coefficient greater than the threshold. A similar process was performed with the 25 samples from the oldest patients using the same list of miRNAs. In this case, a Pearson correlation coefficient greater than or equal to 0.62 differentiates the distributions of the correlations between the miRNAs and those of a random population. In LO-PCa, 33 miRNAs were coexpressed over the threshold and were used for the following analysis.

### miRNA-miRNA interactions to detect hub miRNAs

**Figure 3:**
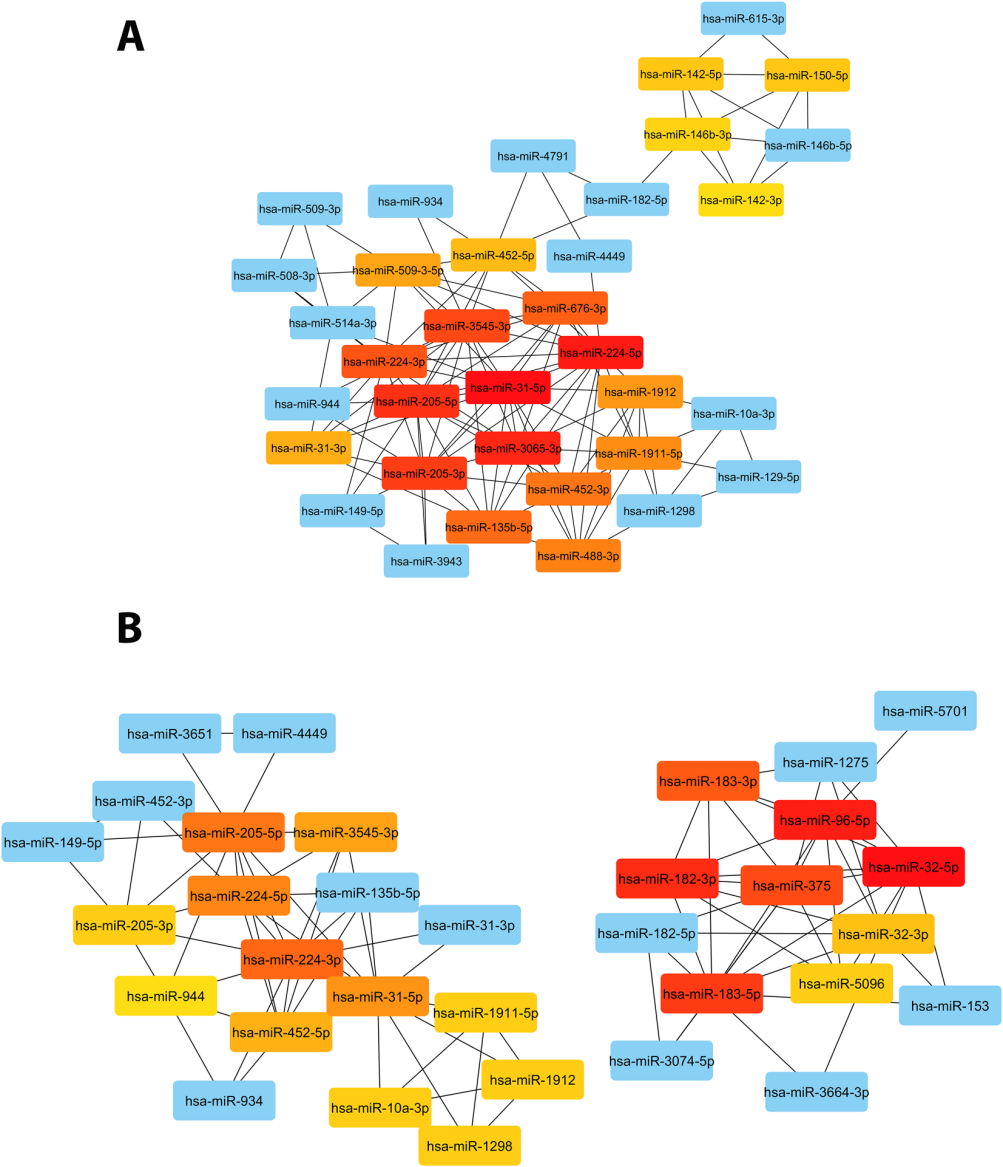
Network analysis identified hub miRNAs using cytoHubba plug-in ranked by MCC. (**A**) EO-PCa. (**B**) LO-PCa. miRNAS with high centrality are noted in red. miRNAS with high-moderate centrality are noted in orange. miRNAS with low-moderate centrality are noted in yellow. miRNAS with low centrality are noted in blue.

The networks calculated were analyzed using Cytoscape. The top 20 nodes ranked by the metrics MCC implemented CytoHubba were chosen^22^. In young and elderly samples, two well-defined networks composed of downregulated and upregulated miRNAs were observed (Fig. 3 and Table 1). In EO-PCa, the network had 35 nodes, one connected component, a clustering coefficient of 0.695, network centralization of 0.250, and an average of 6.97 several partners of neighbors. On the other hand, in LO-PCa, the network had 33 nodes, two connected components, a clustering coefficient of 0.740, network centralization of 0.180, and an average of 5.394 several partners of neighbors. The score of the top 10 hub miRNAs was from 1560 to 7716 in the young network and from 60 to 182 in the old network. In the EO-PCa top 10 miRNAs, all miRNAs were downregulated. However, in the LO-PCa network, the top 6 miRNAs were upregulated, and the following 4 were downregulated. In the comparison between the top 10 and the top group, hsa-miR-3065-3p and hsa-miR-676-3p were exclusively identified in young patients, whereas these genes did not exhibit statistical significance in the elderly population. Additionally, in the top 20, hsa-miR-488-3p was exclusively identified in young patients, but this gene was not statistically significant in elderly patients. In the top 20 miRNAs, nine downregulated miRNAs were present in the two groups (hsa-miR-31-5p, hsa-miR-224-5p, hsa-miR-205-5p, hsa-miR-205-3p, hsa-miR-3545-3p, hsa-miR-224-3p, hsa-miR-1911-5p, hsa-miR-1912, and hsa-miR-452-5p). In addition, only three miRNAs were upregulated in young patients (hsa-miR-150-5p, hsa-miR-142-5p, and hsa-miR-146b-3p). This result is in contrast to elderly patients, where eight miRNAs were upregulated (hsa-miR-32-5p, hsa-miR-96-5p, hsa-miR-182-3p, hsa-miR-183-5p, hsa-miR-375, hsa-miR-183-3p, hsa-miR-32-3p, and hsa-miR-5096). The exclusively upregulated and downregulated hub miRNAs in the young patients were not statistically significant in the elderly patients.

To identify the relevance of the hub miRNAs in the EO-PCa network we used miRNet to perform a KEGG pathway enrichment analysis with the predicted transcriptional targets of the 20 hub miRNAs. The analysis showed that 32 genes in the Prostate Cancer pathway were potentially regulated by the selected miRNAs (Supplementary File 1).

### Assessment of prognostic significance of EO-PCa cohort

**Figure 4:**
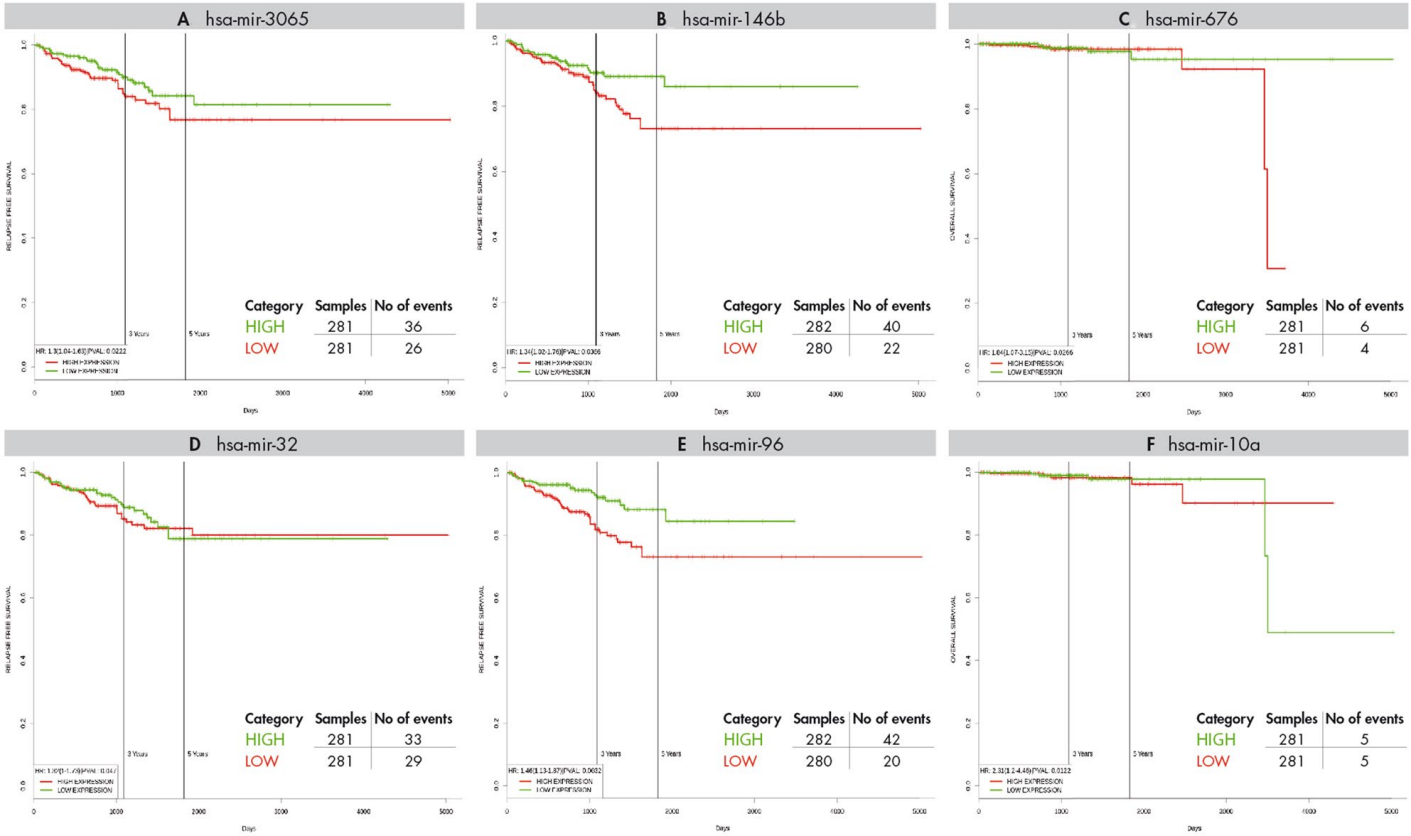
Kaplan–Meier survival plots for overall survival related to hub miRNAs exclusively identified in young patients. The X and Y axes represent survival time (days) and recurrence-free survival (**A** and **C**) or percent survival (**B**), respectively. The analysis was made in PROGmiR V2.

The prognostic significance of the 20 hub miRNAs of the EO-PCa network were analyzed via PROGmir V2, which used the PRAD dataset^23^. Of the 20 hub miRNAs, four had prognostic value using survival data from general PCa. The hub miRNAs that are exclusively dysregulated in EO-PCa were hsa-miR-3065 (Hazard ratio (HR): 1.3) and hsa-miR-146b (HR: 1.34), which are associated with poor relapse‐free survival. In addition, miR-676 (HR: 1.84) was related to poor overall survival. On the other hand, two miRNAs exclusively identified in old patients, namely hsa-miR-32 and hsa-miR-96 (HR: 1.46), were associated with poor relapse‐free survival. In addition, hsa-miR-10a (HR: 2.31) was related to poor overall survival. Figure 4 presents the Kaplan-Meir survival plots and the number of events in each analysis.

### Correlated mRNA genes with hub miRNAs from the EO-PCa coexpression network

To molecularly explain the effects of dysregulation of those miRNAs in EO-PCa, we identified genes that were coexpressed with the hub miRNAs. We used mRNA microarray data generated using the same samples from the same patients who were used to generate the miRNA expression data.

After normalization and batch effect correction of the microarray dataset, six samples were identified as outliers, three in EO-PCa patients and three in LO-PCa patients. Those samples were not included in the additional analysis.

The Pearson coefficients of the correlation of the 20 hub miRNAs in EO-PCa with the genes in the microarray were calculated for the EO-PCa samples and similarly for the LO-PCa samples. A permutation test revealed that correlation coefficients greater than or less than ± 0.614 were statistically significant for EO-PCa samples and correlation coefficients greater than or less than ± 0.673 were statistically significant for LO-PCa samples.

Different numbers of coexpressed genes were identified for each hub miRNAs with numbers ranging from 0 to 167 genes. Supplementary file 2 presents the numbers, names and correlation coefficients of the coexpressed genes for EO-PCa and LO-PCa samples. The most remarkable result was that EO-PCa upregulated hub miRNAs had more coexpressed genes than downregulated miRNAs, and most of these miRNAs exhibited positive correlation coefficients (Supplementary file 2).

To provide a biological meaning of the lists of coexpressed genes, a pathway analysis with the statistically significant coexpressed genes for each hub miRNA was performed. We performed an overrepresentation analyses against the 21 cancer prostate pathways that we collected from Molecular Signatures Database (MSigDB)^24^ and the list of DEGs detected by Ding et al. (Supplementary file 3). We found that the three upregulated miRNAs (hsa_miR_142_5p, hsa_miR_146b_3p and hsa_miR_146b_3p) were coexpressed with the DEGs in EO-PCa versus normal tissue (Ding Early onset prostate cancer 2016)^9^. Two of the upregulated miRNAs were coexpressed with DEGs upregulated in the more aggressive prostate cancers of African-Americans compared with the less aggressive prostate cancers of European-American patients (WALLACE PROSTATE CANCER RACE UP)^25^ (Table 2). Among the downregulated miRNAs, hsa_miR_3545_3p and hsa-miR-224-5p were significantly coexpressed with genes in LIU PROSTATE CANCER DN^26^(Table 2). This pathway was obtained from microarray analysis of 31 PCa samples. The Gleason score was variable, and information on patient age was not provided. Table 3 shows all the genes with significant correlation coefficients for Ding Early onset prostate cancer 2016 and WALLACE PROSTATE CANCER RACE UP.

**Table 2:** Over-representation analysis of co-expressed genes with hub miRNA using EO-PCa data.

hub miRNA	Gene set	Size	Expect	Ratio	Overlap	FDR
hsa_miR_142_5p	WALLACE PROSTATE CANCER RACE UP	277	3.64	4.12	15	1.0889E−06
hsa_miR_150_5p	WALLACE PROSTATE CANCER RACE UP	277	2.32	3.88	9	8.83E−04
hsa_miR_142_5p	Ding Early onset prostate cancer 2016	158	2.08	5.78	12	1.0889E−06
hsa_miR_150_5p	Ding Early onset prostate cancer 2016	158	1.32	6.81	9	1.46E−05
hsa_miR_146b_3p	Ding Early onset prostate cancer 2016	158	6.89	4.93	34	3.12E−13
hsa_miR_3545_3p	LIU PROSTATE CANCER DN	473	5.65	2.30	12	0.015

Only statistically significant associations are shown. Size means the number of genes in the pathway. Expect means the expected number of genes in the pathway co-expressed by chance with the miRNA. Ratio means the additional number of times that there are more genes co-expressed with the miRNA compared with the expected number. Overlap means the number of co-expressed genes that are part of the pathway. FDR is the false discovery rate of the overlap.

**Table 3:** Genes with coefficient of correlation statistically significant for Ding Early onset prostate cancer 2016 and WALLACE_PROSTATE_CANCER_RACE_UP with upregulated hub miRNAs in EO-PCa.

Ding Early onset prostate cancer 2016				WALLACE_PROSTATE_CANCER_RACE_UP		
ID_REF	hsa-miR-142-5p	hsa-miR-150-5p	hsa-miR-146b-3p	ID_REF	hsa-miR-142-5p	hsa-miR-150-5p
ADAMTS1	NS	NS	− 0.639	ADAMDEC1	0.724	0.715
APOE	NS	NS	0.648	**CCR7**	0.695	0.680
C4A	NS	NS	0.664	CD28	0.668	NS
CCDC74B	NS	NS	− 0.694	**CD3D**	NS	NS
CCL19	NS	NS	0.660	CD48	0.657	NS
**CCR7**	0.695	0.680	0.746	CXCL9	0.677	NS
**CD3D**	NS	NS	0.644	DOCK10	0.681	0.693
CD3E	NS	0.651	0.685	GZMK	0.707	0.709
CD3G	0.684	0.684	0.644	IDO1	0.657	NS
CD6	0.641	0.644	NS	IL2RG	0.723	0.712
CD84	0.654	NS	0.675	**IL7R**	0.647	0.663
COL2A1	NS	NS	0.658	ITGB2	0.705	0.640
CP	NS	NS	0.648	**ITK**	0.685	0.659
E2F2	NS	NS	0.681	**MMP9**	0.640	NS
EOMES	0.650	NS	0.673	PLEK	0.655	NS
ERG	NS	NS	0.653	**PTPRC**	0.675	0.689
HIST1H2AI	NS	NS	0.723			
HIST1H2BM	NS	NS	0.701			
HLA-DMB	NS	NS	0.705			
IKZF1	NS	NS	0.716			
**IL7R**	0.647	0.663	0.702			
**ITK**	0.685	0.659	0.681			
LEPREL1	NS	NS	− 0.770			
LTB	0.639	NS	0.661			
MMP7	NS	NS	0.687			
**MMP9**	0.640	NS	0.650			
PDE3B	NS	NS	0.667			
PLP1	NS	NS	− 0.705			
**PTPRC**	0.675	0.689	0.687			
PYHIN1	0.680	0.683	0.649			
SERPINA3	NS	NS	0.709			
SLAMF6	0.726	0.672	0.647			
SLC35F1	NS	NS	− 0.654			
TMEM178	NS	NS	0.671			
UBD	NS	NS	0.767			

Genes in bold are common genes between both pathways.

A similar analysis was performed using the LO-PCa data, demonstrating that none of the miRNAs had more coexpressed genes in the pathways of early onset prostate cancer or more aggressive cancer in African-Americans than expected by chance. Four miRNAs (hsa_miR_31_5p, hsa_miR_205_5p, hsa_miR_224_3p, and hsa_miR_3545_3p) were significantly coexpressed with genes in the pathway LIU PROSTATE CANCER DN^26^ (Supplementary Table 2).

### Assessment of prognostic significance of genes coexpressed with hub miRNAs

**Figure 5:**
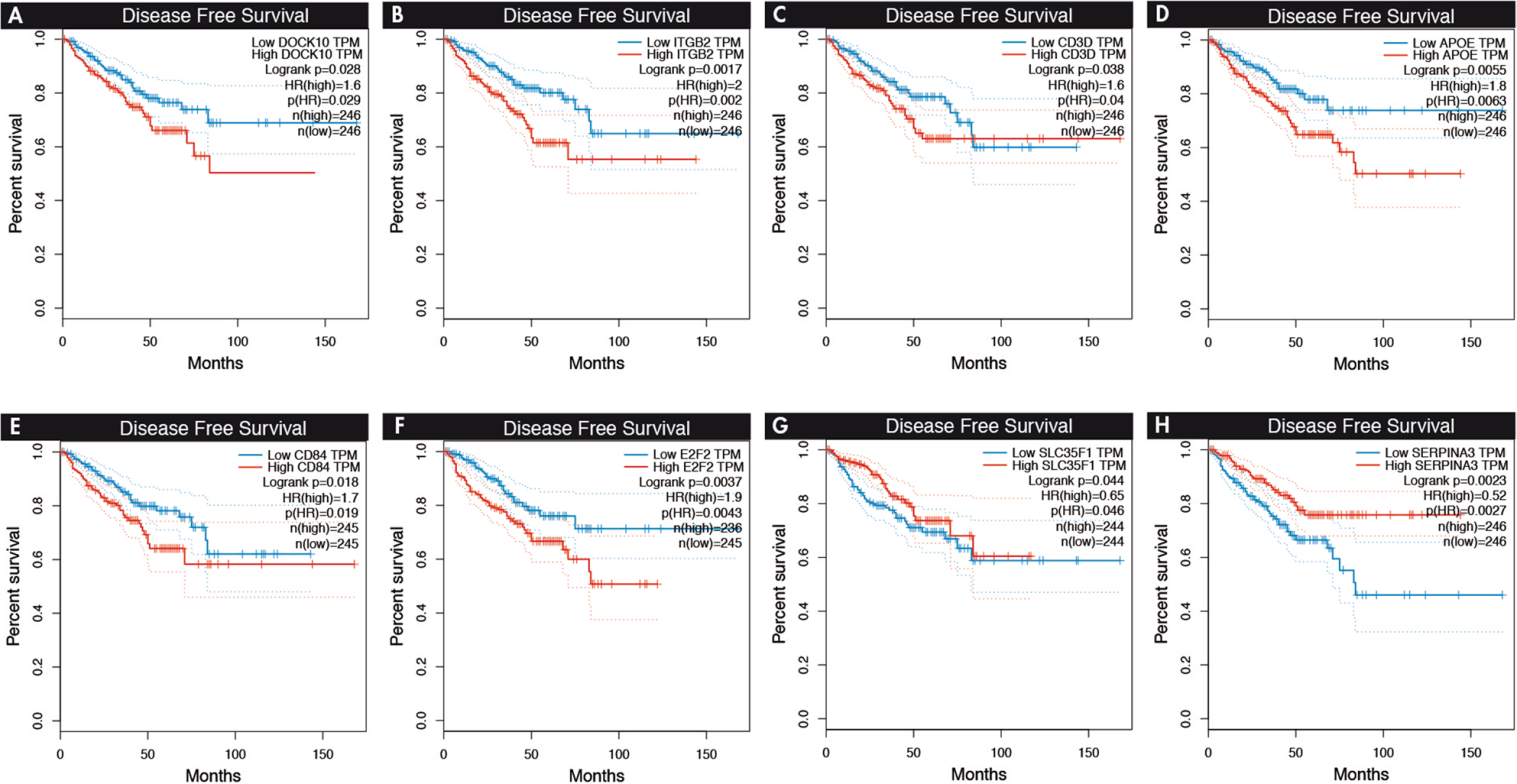
Kaplan–Meier survival plots for overall survival related to target genes correlated with hub miRNAs. The X and Y axes represent survival time (months) and disease-free survival, respectively. The analysis was made in GEPIA.

Using GEPIA^27^, which employed the PRAD dataset, the genes correlated with hub miRNAs that exhibited statistical significance were analyzed (Table 3). Among the upregulated hub miRNAs, two genes that exhibited high expression (DOCK10: HR: 1.6, *p* = 0.02; ITGB2: HR: 2, *p* = 0.001) (Fig. 5) in the Wallace_Prostate_Cancer_Race_Up database were associated with poor disease‐free survival. In the Ding_PlosGenetics2016, the high expression of four genes (CD3D: HR: 1.6, *p* = 0.03; APOE: HR: 1.8, *p* = 0.005; CD84: HR: 1.7, *p* = 0.01; E2F2: HR: 1.9, *p* = 0.003) was associated with poor disease‐free survival, and the low expression of two genes (SLC35F1: HR: 0.65, *p* = 0.04; SERPINA3: HR: 0.52, *p* = 0.002) served as protective factors. In the hub of downregulated miRNAs, the low expression of FBXO17 served as a protective factor (HR: 0.63, *p* = 0.03) of disease‐free survival.

## Discussion

EO-PCa is a subtype of PCa, which is currently receiving high interest due to its impact on clinical behavior and pathobiological differences with the “classical” or elderly PCa (LO-PCa). In this study, novel data analysis was performed using transcriptomic data from patients with EO-PCa who were < 45 years old and LO-PCa who were 71 to 74 years old. The data analyzed were generated by Ding et al. using 49 PCa patients^9^. The tumor samples were GS 7 (3 + 4) and grade T2 (T2a or T2c). Samples were obtained from different ethnic groups, including 88% whites, 4% African-Americans, 4% Hispanics and 4% Asians. We identified 55 miRNAs DE in EO-PCa, including 28 upregulated and 27 downregulated. In addition, 26 of these genes were exclusively dysregulated in EO-PCa. Using an overrepresentation analysis with the predicted targets genes of the miRNAs DE, we identified several pathways commonly dysregulated between EO-PCa and LO-PCa. These pathways are related to adherence junctions, cell cycle and p53 signaling. In addition, the neurotrophin signaling pathway was identified as dysregulated, and members of this pathway are expressed in PCa, i.e., trk receptors and neurotrophins (NGF, BDNF, and/or NT-3).^28^^.^ Strikingly, in upregulated miRNAs in elderly patients, the lysine degradation pathway exhibited statistical significance. Lysine modification is associated with carcinogenesis in different types of tumors^29^.

Among the hub miRNAs exclusively deregulated in EO-PCa without significance in LO-PCa, three were downregulated (hsa-miR-3065-3p, hsa-miR-676-3p, and hsa-miR-488-3p), and three were upregulated (hsa-miR-150-5p, hsa-miR-142-5p, and hsa-miR-146b-3p). The role of hsa-miR-3065-3p and hsa-miR-676-3p in PCa is unknown, but reduced expression of these genes is observed in tumors, such as esophageal squamous cell carcinoma^30^, hsa-3065-3p in clear cell renal cell carcinoma^31^, and hsa-miR-676-3p in breast cancer cell lines^32^. In survival analysis, hsa-miR-3065 was associated with poor relapse‐free survival, and hsa-miR-676 was related to poor overall survival. In the PCa cell lines (LNCaP and C4-2B), hsa-mir-488-3p inhibits the androgen receptor, blocks proliferation and induces apoptosis^33^. Among the exclusively upregulated hub miRNAs in EO-PCa, hsa-miR-142-5p and hsa-miR-150-5p had been described as deregulated^34–37^. The last may act as antitumor miRNA targeting SPOCK1^35^. hsa-miR-150 has been reported as upregulated with a role in proliferation and invasion by targeting p53^36^, and its expression is associated with poor overall survival (HR: 1.87, CI: 1.19–2.94)^37^. In addition, Ding et al.^9^ found that hsa-miR-146b-3p exhibited the highest level of overexpression in young PCa patients. However, the specific role of hsa-miR-146b-3p in PCa its unknown, it was associated with poor relapse‐free survival in the survival analysis (Fig. 4). hsa-miR-146b-3p is member of the miR-146 a/b family. hsa-miR-146a is dysregulated in PCa and other tumors. In androgen-independent PCa, its downregulation is involved in apoptosis through regulation of the ROCK/Caspase 3 pathway^38,39^.

To understand how dysregulation of hub miRNAs modulates the normal behavior of prostatic tissue, we searched the targets genes of each hub miRNAs. The number of potential target genes identified was 112 for hsa-miR-3065-3p, 51 for hsa-miR-676-3p, 131 for hsa-miR-488-3p, 534 for hsa-miR-150-5p, 226 for hsa-miR-142-5p, and 56 for hsa-miR-146b-3p. These hub miRNAs are annotated as members in different KEGG pathways that are relevant in PCa biology. For example, regulatory targets of hsa-miR-3065-3p, such as GSK3B (Glycogen Synthase Kinase-3), are involved in apoptosis, cell cycle, DNA repair, tumor growth, invasion, and metastasis pathways. In recent years, it has become a targeted gene for therapy^40^. In the progression to androgen-independent PCa, GSK3B may act with PTEN^41^ as a positive regulator of androgen receptor transactivation and growth independent of the Wnt/β-catenin pathway^42^. hsa-miR-3065-3p and hsa-miR-488-3p are related to p53, which is a very relevant gene that is mutated in greater than half of all cancers and is associated with progression in PCa^43^. hsa-miR-3065-3p is repress by mutant p53^44^, and hsa-miR-488-3p activates the p53 pathway through suppressing ZBTB2^45^. On the other hand, C-terminal Binding Protein 1 (CTBP1) is a target gene of hsa-miR-676-3p. This gene is a transcriptional corepressor of tumor suppressors genes involved in cell death, and dysregulated expression of this gene is associated with PCa progression^46^. Platelet-derived growth factor receptors-β (PGDFR-β) is regulated by hsa-miR-488-3p and hsa-miR-146b-3p), which are key regulators of cell growth and division^47^. In PCa, PGDFR-β is expressed in the early stage of the disease^48^. Its activation is associated with the loss of PTEN^49^, and high PGDFR-β expression is associated with prostate cancer recurrence^50^.

Among the upregulated hub miRNAs, several target genes involved in the KEGG PCa pathway were identified. The targets of hsa-miR-150-5p in this pathway include CDK2, EP300, and TP53. CDK2 is a key regulatory protein involved in cell cycle arrest upon DNA damage^51^. It is upregulation is associated with PCa progression, and it is a probable novel target gene in treatment^52^. hsa-miR-142-5p is modulator of important genes involved in the pathogenesis of PCa, including Cyclin D1 (CCND1), MAPK1, and PTEN. CCND1 is associated with aggressiveness^53^. MAPKs are serine/threonine kinases that mediate intracellular signaling associated with a variety of cellular activities, such as cell proliferation. In PCa, MAPKs are involved in apoptosis, survival, metastatic potential, and androgen-independent growth^54^. Additionally, PTEN is the most commonly lost tumor suppressor gene in primary disease. In most cases with PTEN loss, the gene is lost by genomic deletion. The loss of PTEN is associated with prostate tumor aggressiveness, progression, and poor prognosis (reduced disease-specific survival)^55^.

Given the limited number of studies on the effects of the hub miRNAs in the pathogenesis of PCa, we performed a correlation analysis based on the expression of hub miRNAs and all the genes in the genome in patients with EO-PCa. The overrepresentation analysis of the genes with significant correlations among hub miRNAs in the EO-PCa network revealed that the three upregulated hub miRNAs were significantly coexpressed with the Ding Early-onset prostate cancer 2016 pathway: two of the upregulated hub miRNAs were coexpressed with Wallace_Prostate_Cancer_Race_Up and one downregulated hub miRNA was coexpressed with LIU PROSTATE CANCER DN (Table 2). In the primary analysis of the data used in the present study, Ding et al.^9^ reported differential expression of genes annotated in immunological pathways in the age:tissue interaction analysis (B Cell Development, iCOS-iCOSL Signaling in T Helper cells, CD28 Signaling in T Helper Cells, Primary Immunodeficiency Signaling, Calcium-induced T Lymphocyte Apoptosis), including genes such as complement family genes, immune-cell surface antigens, chemokines, interleukin receptors, natural killer cells and extracellular matrix remodeling genes. Moreover, the Wallace_Prostate_Cancer_Race_Up^25^ dataset was generated from the comparison of gene expression profiles of PCa from 33 African-American patients with 36 European-American patients. The genes in this pathway are involved in immune response, stress response, cytokine signaling, and chemotaxis pathways. Several known metastasis-promoting genes, including autocrine mobility factor receptor, CXCR4 (chemokine (C-X-C motif) receptor 4), and MMP9, were more highly expressed in tumors from African-Americans than European- Americans. The expression profiles of two upregulated hub miRNAs, namely hsa-miR-150-5p and hsa-miR-142-5p, were correlated with the Wallace_Prostate_Cancer_Race_Up dataset.

The genes shared in Wallace and Ding pathways and statistically correlated with upregulated hub miRNAs (hsa-miR-150-5p and hsa-miR-142-5p) are genes involved in the immunology response, such as CCR7, IL7R, ITK, PTPRC, MMP9, APOE, CCL19, and CD3D. These genes were upregulated in PCa of African-American patients and in EO-PCa^25^. CCR7 and MMP9 are genes associated with PCa progression and metastases^56–58^. MMP-9 is involved in several hallmarks of PCa progression, such as proliferation, angiogenesis, epithelial to mesenchymal transition, apoptosis, and metastasis^59^. MMP-9 expression is associated with the risk of PCa (OR = 7.91; 95% CI: 5.27–11.89; *P* < 0.00001)^60^. A primary PCa cell line derived from an African–American patient (E006AA) exhibited increased MMP9 expression compared to other studied cell lines (LNCaP, C4-2, and MDAPCa2b)^61^^.^ CCR7 is a chemokine receptor that is associated with lymph node metastasis in other tumors, such as breast cancer^62^, non-small cell lung cancer^63^, and gastric carcinoma^64^. The CCR7 ligand CCL21 is expressed selectively in high endothelial venules at the entry point into the lymph node and promotes cancer progression^56,62^^.^ Polymorphisms in CCR7 (rs3136685) are present in African–American PCa patients^65^. IL7R and IL7 are highly expressed in PCa and are associated cancer cell invasion and migration probably by activating the AKT/NF-κB pathway and upregulating MMP-3 and MMP-7 expression^66^.

All the coefficients of correlation among the upregulated hub miRNAs and the mRNAs genes were positive. It could be counterintuitive that the upregulation of a specific miRNA causes the up-regulation of a specific mRNA. We proposed that it may explained by the activation of the transcription of target genes by binding of miRNAs with reverse complementary sequences in promoter regions of genes^14^. For example, overexpression of hsa-miR-205 increases IL-35 and IL-24 expression^67^. In PCa cells (DU145 and PC3), overexpression of miR-3619-5p induces CDK1N1 gene expression via direct interaction with the promoter region^68^. On the other hand, one gene target can be targeted by several miRNAs^69^. Therefore, the targets of this gene are potentially regulated by miRNAs DE. Thus, it is necessary study the regulation of this gene’s targets.

In the analysis of overall survival of genes with high expression in Wallace_Prostate_Cancer_Race_Up^25^, high expression of DOCK10 and ITGB2 was associated with poor prognosis. Both genes are coexpressed with hsa-miR-150-5p and hsa-miR-142-5p. The roles of these genes in PCa are unknown. In cancer, DOCK10 (Dedicator Of Cytokinesis 10) is involved in the regulation of the epithelial to mesenchymal transition^70^. ITGB2 (Integrin beta-2 (CD18)) combines with integrin alpha to form the integrin lymphocyte function-associated antigen-1 (LFA-1). This gene is involved in tumor growth and metastasis^71^.

A limitation of this study was the high homogeneity of the patients, who all had Gleason scores of 7 (3 + 4). Patients with low-grade PCa have different molecular characteristics, clinical behavior and treatment^72^. However, the expression profile of miRNAs in low-grade PCa does not exhibit significant differences. Walter B et al.^73^ compared miRNA expression in 26 patients with low-grade and 15 patients high-grade PCa. The results did not reveal any specific signatures to differentiate the two groups. Another limitation involves the survival analyses of DE miRNAs and DEGs because the data were obtained from TCGA database. In this analysis, we could not perform separate evaluations for EO-PCa and LO-PCa.

In conclusion, this is the first study that analyzed the expression of miRNAs in EO-PCa and LO-PCa patients using network analysis. Connections among miRNA expression, target genes, and molecular pathways for EO-PCa and LO-PCa were identified. Furthermore, specific miRNAs with clinical significance in young patients may explain molecular differences, and the different biological processes in young and elderly patients were identified. In addition, we found coexpression of genes and hub miRNAs that play important roles in PCa progression and metastasis and genes associated identified in Afro-American PCa patients. Most of these genes are involved in the immunology response. As a recommendation, the constructed network of biomarkers should be further assessed in EO-PCa, and candidate miRNAs and gene targets should be validated for patient diagnosis and prognosis.

## Materials and methods

### Data selection

In June 2019, an advance search was performed to identify studies that analyze miRNA expression in EO-PCa patients. The sources used included PubMed and the National Center for Biotechnology Information (NCBI) GEO database (https://www.ncbi.nlm.nih.gov/geo/). The keywords 'young OR early-onset AND prostate cancer' were used in the search. The results were limited to *Homo sapiens* as the organism and expression profiles were determined using array dataset types.

The inclusion criteria for the systematic review were (1) miRNA expression was assessed in prostate tissue of young and elderly PCa patients in the same dataset, (2) raw data were available, and (3) data passed quality control. Two reviewers performed an eligibility assessment by screening titles and abstracts from the publications. Subsequently, the articles that did not meet the eligibility criteria were rejected. Additionally, we searched The Cancer Genome Atlas (TCGA); however, we could not use these data because the database only contained matched tumor and normal data from three patients diagnosed with PCa under the age of 55 years.

### Preprocessing and identification of differentially expressed miRNAs

Raw counts of miRNAs from 49 patients with prostate cancer diagnoses were downloaded from GSE89193 and deposited in the Gene Expression Omnibus (GEO) database (https://www.ncbi.nlm.nih.gov/geo/query/acc.cgi?acc=GSE89193). The data included 25 elderly men (ages 71–74 years) and 24 young men (ages 38–45 years). For each patient, the tumor and a standard region of the prostate were analyzed. Samples were sequenced on the Illumina HiSeq 2.500 platform (https://www.illumina.com/systems/sequencing-platforms/hiseq-2500.html).

Raw count miRNA data were normalized using the trimmed mean of the M-value (TMM) method through the EdgeR package^74^. Because the original data were sequenced in two batches^9^, this nonbiological variability source was corrected using nonparametric Bayesian statistics methodology using the sva package.^75^. The extent of the batch effect correction was assessed by principal component analysis.

The samples were assigned to two different experimental groups to identify the differentially expressed miRNAs in LO-PCa (71–74 years old) and EO-PCa (38–45 years old). miRNA expression levels were compared between tumor and normal samples in each group. The statistical significance level for this study was calculated using the Limma statistics package (Linear Models for Microarray and RNA-Seq Data)^76^. Limma uses a linear modelling to detect differentially expressed genes. The fold change was calculated, and the statistical significance was adjusted for multiple comparisons (False Discovery Rate (FDR)).

### Weighted gene coexpression networks analysis (WGCNA)

The total levels of differentially expressed miRNAs between normal and tumor samples in older and young patients were collected in one list. Two coexpression networks were developed using this gene list. The first list was generated for the young samples, and the second list was generated for the older group. First, the similarity matrix was calculated by identifying the Pearson correlation coefficients of the expression levels for the samples based on all possible gene pairs. Then, the similarity threshold was calculated with the adjacency function, which was established according to the unique characteristics of each similarity matrix^77^. The method developed by Elo was used to select the threshold^78^. This method compared the tau values for the network grouping coefficient (Co) with that expected for a random network (Cr). It uses the clustering coefficient of the real graph in comparison to a rando graph. The threshold for significant similarities is chosen so that the obtained real graph is scale free. Finally, the adjacency matrix (2 × 2) of the network was established and allowed the representation of binary relationships. In this case, a pair of genes that exhibit coordinated gene expression activity (coexpression) is indicated by (1); otherwise, a (0) is reported. All WGCNA analyses were performed in an R unique environment using statistical functions (https://www.r-project.org/).

### Detection of hub miRNAs

The hub miRNAs were identified through network analysis using Cytoscape and its plugin (CytoHubba). This plugin accurately identifies hub genes by 12 topological analysis methods. For this study, the Maximal Clique Centrality (MCC) method proposed by CytoHubba was used; recently, this method exhibits improved performance to capture essential targets in the top rank list in both high- and low-grade PCa^22^. In addition, MCC helped to identify the top 20 hub miRNAs. On the other hand, the network analyzer plugin was used to recognize the network parameters.

### Functional annotations of hub miRNAs

The miRNet database to identify target genes (https://www.mirnet.ca/) was used to facilitate the interpretation of biological mechanisms related to hub miRNAs. This tool integrates data from eleven different miRNA databases: TarBase, miRTar-Base, miRecords, miRanda, miR2Disease, HMDD, PhenomiR, SM2miR, PharmacomiR, EpimiR, and starBase^79^. The following information was provided for miRNet analysis: organism name (*H. sapiens*), ID type (miRBASE ID), and tissue origin (Not specified). No degree or betweenness filter was used for network visualization. Additionally, miRNet was applied to identify biological pathways and processes, molecular functions, and cellular components that are statistically enriched for the corresponding miRNA target genes. For the functional evaluation of the miRNAs, the Kyoto Encyclopedia of Genes and Genomes (KEGG) enrichment analyses were conducted in mirNet. Only statistically significant annotation categories (*P* value < 0.05) were retained.

### Survival analyses of miRNAs with dysregulated expression

PROGmiR V2 is an online free tool and is available at https://www.compbio.iupui.edu/progmir. This program combines the prognostic data of miRNAs for different types of cancers from TCGA dataset. This tool was used to compare the overall, relapse-free, and metastasis-free survival of prostate adenocarcinoma patients with DE of miRNAs in young and old cohorts. It also divides samples based on high and low expression and calculates the hazard ratio (HR) with relative confidence intervals (CI) and *P* values for the proportional hazards model^23^.

### Determination of mRNA expression levels of all genes in normal and tumor tissues from EO-PCa and LO-PCa for the diagnosis of prostate cancer

The level expression of mRNAs from cancer and normal tissues from EO-PCa and LO-PCa patients were downloaded from the same dataset GSE88808 available in the GEO OMNIBUS database (https://www.ncbi.nlm.nih.gov/geo/query/acc.cgi?acc=GSE88808).

These data were generated in parallel with the miRNA dataset. The same patients and same tissues were used to identify the level expression of mRNAs and miRNAs. A detailed description of the RNA obtention and determination of mRNA expression level are provided in the primary paper^9^. In summary, total RNA was extracted from approximately 5 mg of unsectioned formalin-fixed paraffin-embedded core samples using the RecoverAll Total Nucleic Acid Isolation kit (Life Technology, Inc.). The Illumina HumanHT-12 WG-DASL V4.0 expression beadchip was used for mRNA expression profiling of 29,000 genes in the human genome. The levels of intensity were normalized using the quantile normalization method^80^. The batch effects secondary to different times of hybridization were corrected using the empirical Bayes methods as is implemented in the ComBat in sva package^81^.

Identification of outliers was performed using the Pearson correlation measurements of the level expressions of all genes in the microarray between all the samples. Samples with correlation coefficients less than 0.9 compared with the other samples were excluded from additional analyses.

### Analysis of the correlation of the expression of hub miRNAs with the expression of genes involved in the pathogenesis of prostate cancer

The hub miRNAs in the EO-PCa coexpression network were included in additional correlation analyses to determine whether they were coexpressed with genes involved in prostate cancer.

First, we collected the 183 differentially expressed genes (DEGs) identify by Ding et al.in the primary analysis of the mRNA expression data^9^; they identified differences in tumor vs. normal tissues between samples from young and old patients. We refer to this list of genes as Ding Early-onset prostate cancer 2016.

Second, the Molecular Signatures Database (MSigDB)^24^ was interrogated to collect the pathways related to prostate cancer. In total, 22 different pathways were identified (Supplementary file 3). This collection of pathways represents the state of knowledge about transcriptomic modifications between tumor tissues compared with normal tissues from prostate cancer patients (19 pathways) and tumor samples from African-American compared with European-American patients with primary prostate cancer^25^.

Third, Pearson correlation coefficients were calculated among the expression profiles of selected hub miRNAs and mRNA expression levels from all the genes in the Illumina microarray. We initially performed correlation analysis only for young or old samples. Using a permutation test, the confidence intervals were calculated. Correlations coefficients with *P* values less than 0.002 were selected as statistically significant.

Finally, overrepresentation analyses were performed using the hypergeometric test as implemented in WebGestalt^82^. For each selected hub miRNA, statistically significant coexpressed genes were interrogated against the genes in the 22 prostate cancer pathways to identify whether more (overrepresentation) genes coexpressed with miRNAs are present than expected by chance.

### Survival analyses of genes coexpressed with hub miRNAs

Gene Expression Profiling Interactive Analysis (GEPIA; https://www.gepia.cancer-pku.cn)^27^ was used to calculated disease‐free survival and overall survival between DEGs coexpressed with hub miRNAs. The lower and upper 50% of gene expression levels were set as the standard for analysis. The confidence interval was 95%. High and low expression genes are represented in red and blue, respectively. Log-rank test results with *P* < 0.05 were regarded as statistically significant.

## Supplementary information

Supplementary information 1

Supplementary information 2

Supplementary information 3

Supplementary information 4

Supplementary information 5
