# Supplementary material for: Identification of candidate miRNAs in early-onset and late-onset prostate cancer by network analysis
Source: Sci Rep. 2020 Jul 23;10:12345. doi: 10.1038/s41598-020-69290-7 (PMC7378055; doi:10.1038/s41598-020-69290-7)
Supplement: Supplementary file 1 — Supplementary information 1 [file 41598_2020_69290_MOESM1_ESM.pdf]

Identification of candidate miRNAs in early-onset and late-onset prostate cancer by network analysis

Rafael Parra-Medina [1,2,3], Liliana López-Kleine [4] , Sandra Ramirez-Clavijo [1], César Payán-Gómez[1*]

1. Faculty of Natural Sciences. Universidad del Rosario, Bogotá, Colombia
2. Research Institute, Department of Pathology. Fundación Univeristaria de Ciencias de la Salud. Bogotá, Colombia
3. Pathology deparment. Instituto Nacional de Cancerología, Bogotá, Colombia
4. Department of Statistics, Faculty of Science, Universidad Nacional de Colombia, Bogotá, Colombia.

Supplementary table 1. miRNAs DE exclusive in EO-PCa, LO-PCa and share (log FC ≥ 1 or ≤ 1, and adj.P.Val <0.01).

miRNAs exclusive in EO-PCa	Log FC	miRNAs exclusive in LO-PCa	Log FC	miRNAs share in young and old	Log FC in young	Log FC in old
**Upregulated**						
hsa-miR-4791	1.86	hsa-miR-4417	1.88	hsa-miR-3687	2.24	3.08
hsa-miR-146b-3p	1.57	hsa-miR-1973	1.81	hsa-miR-615-3p	2.23	3.28
hsa-miR-146b-5p	1.37	hsa-miR-4485-3p	1.8	hsa-miR-1275	1.97	1.58
hsa-miR-183-3p	1.34	hsa-miR-3074-5p	1.62	hsa-miR-4449	1.84	1.74
hsa-miR-1248	1.21	hsa-miR-147b	1.56	hsa-miR-153-3p	1.78	2.17
hsa-miR-183-5p	1.17	hsa-miR-32-5p	1.37	hsa-miR-190b-5p	1.74	1.47
hsa-miR-142-5p	1.14	hsa-miR-375	1.29	hsa-miR-663b	1.43	1.44
hsa-miR-3653-3p	1.12	hsa-miR-3664-3p	1.23	hsa-miR-5701	1.27	1.05
hsa-miR-150-5p	1.12	hsa-miR-5096	1.81	hsa-miR-5690	1.24	1.44
hsa-miR-92a-1-5p	1.11	hsa-miR-4461	1.09	hsa-miR-3651	1.23	1.29
hsa-miR-142-3p	1.07	hsa-miR-182-3p	1	hsa-miR-182-5p	1.22	1.1
hsa-miR-3607-3p	1.03			hsa-miR-4517	1.2	1.64
hsa-miR-1268a	1			hsa-miR-32-3p	1.15	1.1
				hsa-miR-96-5p	1.14	1.22
				hsa-miR-4284	1.05	1.43
**Downregulated**						
hsa-miR-1298-5p	-2.76	hsa-miR-509-3p	-1.82	hsa-miR-205-5p	-2.8	-2.33
hsa-miR-1911-5p	-2.58	hsa-miR-452-5p	-1.01	hsa-miR-514a-3p	-2.51	-2.05
hsa-miR-509-3-5p	-2.23	hsa-miR-452-3p	-1	hsa-miR-508-3p	-2.32	-2.45
hsa-miR-1912	-2.02			hsa-miR-3545-3p	-2.51	-1.83
hsa-miR-3943	-1.88			hsa-miR-205-3p	-2.28	-1.94
hsa-miR-31-3p	-1.56			hsa-miR-944	-2.21	-1.99
hsa-miR-129-5p	-1.47			hsa-miR-873-5p	-2.15	-2.18
hsa-miR-488-3p	-1.37			hsa-miR-184	-2.14	-2.59
hsa-miR-676-3p	-1.25			hsa-miR-31-5p	-2.02	-1.82

hsa-miR-10a-3p	-1.12			hsa-miR-187-3p	-1.95	-2.41
hsa-miR-338-5p	-1.12			hsa-miR-135b-5p	-1.95	-1.71
hsa-miR-3065-3p	-1.08			hsa-miR-934	-1.48	-1.79
hsa-miR-149-5p	-1.08			hsa-miR-224-5p	-1.09	-1.42
				hsa-miR-224-3p	-1.05	-1.07
