# Supplementary material for: Identification of candidate miRNAs in early-onset and late-onset prostate cancer by network analysis
Source: Sci Rep. 2020 Jul 23;10:12345. doi: 10.1038/s41598-020-69290-7 (PMC7378055; doi:10.1038/s41598-020-69290-7)
Supplement: Supplementary file 2 — Supplementary information 2 [file 41598_2020_69290_MOESM2_ESM.pdf]

Identification of candidate miRNAs in early-onset and late-onset prostate cancer by network analysis

Rafael Parra-Medina [1,2,3], Liliana López-Kleine [4] , Sandra Ramirez-Clavijo [1], César Payán-Gómez[1*]

1. Faculty of Natural Sciences. Universidad del Rosario, Bogotá, Colombia
2. Research Institute, Department of Pathology. Fundación Univeristaria de Ciencias de la Salud. Bogotá, Colombia
3. Pathology deparment. Instituto Nacional de Cancerología, Bogotá, Colombia
4. Department of Statistics, Faculty of Science, Universidad Nacional de Colombia, Bogotá, Colombia.

Supplementary Table 2. Over-representation analysis of co-expressed genes with hub miRNA using LO-PCa data.

hub miRNA	Gene Set	Size	Expect	Ratio	Overlap	FDR
hsa_miR_31_5p	LIU_PROSTATE_CANCER_DN	473	1.98	3.54	7	0.003
hsa_miR_205_5p	LIU_PROSTATE_CANCER_DN	473	4.81	2.91	14	1.16E-04
hsa_miR_224_3p	LIU_PROSTATE_CANCER_DN	473	7.07	2.69	19	1.93E-05
hsa_miR_3545_3p	LIU_PROSTATE_CANCER_DN	473	9.33	2.25	21	4.73E-04

Only statistically significant associations are shown. Size means the number of genes in the pathway, Expect means the expected number of genes in the pathway co-expressed by chance with the miRNA, Ratio means the additional number of times that there are more genes co-expressed with the miRNA compared with the expected number, Overlap means the number of co-expressed genes that are part of the pathway. FDR is the false discovery rate of the overlap.
